# Lived experience peer support programs for suicide prevention: a systematic scoping review

**DOI:** 10.1186/s13033-020-00396-1

**Published:** 2020-08-12

**Authors:** Marisa Schlichthorst, Ingrid Ozols, Lennart Reifels, Amy Morgan

**Affiliations:** 1 Centre for Mental Health, School of Population and Global Health, the University of Melbourne, Parkville, Australia; 2 mh@work (Mental Health at Work), Melbourne, Australia

**Keywords:** Peer-support, Peer-led, Suicide, Prevention, Literature review, Scoping review

## Abstract

**Background:**

Peer-led support models have gained increasing popularity in suicide prevention. While previous reviews show positive effects of peer-led support for people with mental health problems and those bereaved by suicide, little is known about the types of lived experience peer support programs in suicide prevention and whether these are effective in improving the health and wellbeing of people at risk of suicide. The aim of this paper is to provide an overview of peer support programs that aim to reduce suicidality and are led by people with lived experience of suicide.

**Method:**

We conducted a systematic scoping review, involving a search of three academic (Medline, PsycINFO, Embase) and selected grey literature databases (Google Scholar, WHO Clinical Trials Registry) for publications between 2000 and 2019. We also contacted suicide prevention experts and relevant internet sites to identify peer support programs that exist but have not been evaluated. The screening of records followed a systematic two-stage process in alignment with PRISMA guidelines.

**Results:**

We identified 8 records accounting for 7 programs focussed on peer-led support programs in suicide prevention. These programs employed a range of different designs and included a variety of settings (schools, communities, rural and online). Only 3 of the 7 programs contained data on effectiveness. With the small number of eligible programs the findings from this review are limited and must be interpreted with caution.

**Conclusions:**

Despite the increased focus of policymakers on the importance of peer support programs in suicide prevention, our scoping review confirms an evidence gap in research knowledge regarding program design, implementation, and effectiveness. More rigour is required in reporting peer-led support initiatives to clarify the underlying definition of peer support and lived experience and to enhance our understanding of the types of current peer support programs available to those experiencing suicidality. Further, we need formal and high-quality evaluations of peer support suicide prevention programs led by people with lived experience to better understand their effectiveness on participant health across different settings and delivery modalities and to allow for comprehensive systematic reviews and meta-analysis in future.

## Background

Peer support is a subjective and context specific relationship which is based on lived experience, sharing common life experiences, circumstances, situations and values [1]. It is generally viewed as a “system of giving and receiving help, founded on key principles of respect, shared responsibility and mutual agreement of what is helpful” [2]. This mutual experience creates a deep, holistic understanding where people are able to ‘be’ with each other, without the constraints of a hierarchical (expert/patient) relationship [2] and can focus on the understanding of another’s situation empathically through the shared experience of emotional and psychological pain which can aid recovery [3]. Peer support programs have been shown to offer alternative support options in crisis and care, and an effective strategy to engage with people that traditional health services fail to reach [4].

Aiming to address restrictive psychiatric and mental health care models in the late 18th century, the ex-patient/psychiatric survivor movement advocated for mutual support, user-led activities, reduction of marginalisation and stigma and civil rights for mental health patients [5, 6]. As such the interest in peer support by health care services and research was first focussed on mental health conditions and in line with a greater focus on recovery-based and consumer-focussed care in mental health [3]. Since the early 1990s support has come from government and policy agencies and advocacy groups for building a peer support workforce in health care across a broad range of health conditions [7–9]. More recently Government agencies call to incorporate peer support models within conventional health services for recovery and expand the peer support workforce [10, 11], therefore encouraging new services to be offered to people at risk of suicide.

As such, peer-led mental health support programs both in community and services settings have steadily grown and some evidence for their effectiveness has emerged [12]. For example, studies found that peer support can improve empowerment and hope for recovery for people with severe mental health conditions [13, 14], and reduce mental health symptoms for those individuals with severe mental health conditions (e.g. schizophrenia and clinical depression) [15].

Similarly to the recovery pathway from mental health issues, using peer specialists in suicide prevention may be crucial for constructive coping, support, empowerment, hope and rediscovering meaning in life through the experience of someone who survived suicide [16]. Some evidence on effectiveness is also available for postvention programs for people bereaved by suicide. Summarised in recent literature reviews, peer support programs were effective in reducing grief symptoms, improving psychosocial and suicide related outcomes, and increasing personal growth and well-being in bereaved suicide survivors [17–19]. A few qualitative studies also provide insight into how peer support can positively impact on recovery from suicidality through experiencing mutual understanding, non-judgemental environments and acceptance [16, 20, 21]. However, little is known about what types of peer support models exist for people who experience suicidality and whether these are effective in reducing suicidality [22]. This knowledge gap is at contrast with the growing recognition and presence of peer support programs in health service delivery today.

Addressing this knowledge gap, we undertook a systematic scoping review of literature on peer-led suicide prevention programs with a focus on reducing suicidality in individuals and supporting recovery from suicidality. Our aim was to identify what types of peer-led suicide prevention support programs exist and investigate whether these have been evaluated on their effectiveness to reduce suicide risk. For this review peer support was defined as a suicide prevention program or initiative that was delivered by peers with a lived experience of suicide to people experiencing suicidality or having personal history of suicidality in a formal or informal manner. Hereby formal delivery refers to a specifically designed service or program while informal delivery means an organically grown support initiative mostly found in peer support groups [10]. Lived experience is highlighted as essential in suicide prevention as it follows the rationale of added benefit of personal experience in recovery [8].

## Methods

This scoping review was designed following a methodological framework for scoping studies developed by Arksey and O’Malley’s in 2005 and further revised by Levac et al. in 2010 [23, 24]. The review process followed the recommended five stages: identifying the research question; identifying relevant studies; selecting studies; charting the data; and collating and summarising findings. We included key stakeholder consultations as part of our grey literature search strategy, which is described in detail below.

This review was conducted in accordance with the PRISMA recommendations for systematic reviews [25] and the review protocol was registered with PROSPERO (CRD42018109620).

### Identifying the research question

A research team was convened consisting of three researchers in field of suicide prevention (MS, AM, LR) and one researcher with lived experience and consultancy roles in mental health and suicide related peer-support (IO). Using a co-design methodology, the team met to discuss the purpose of the review and was guided by IO’s experience in the peer support sector of suicide prevention in developing the review protocol and research questions [26, 27].

Two exploratory research questions were developed: What types of suicide prevention peer-support programs delivered by people with lived experience currently exist? What do we know about their effectiveness? These broad questions allow us to generate an overview of research undertaken on this topic and to identify where the research gaps in peer-led support programs in suicide prevention lie.

### Identifying relevant studies

We undertook a systematic search for peer reviewed articles, a search of grey literature databases, and a website search and expert consultations to identify eligible programs. A systematic search of the literature was conducted for articles published between 2000 and 2019 using Medline (PubMed), PsycINFO (OVID interface), and EMBASE (OVID interface). Bibliographies of previous systematic reviews and included papers were also searched. Grey literature was searched to include research that had not been peer-reviewed, including Google Scholar and the World Health Organization (WHO) Clinical Trials Registry (limited to a 5-year period from 2013 to 2018). To identify existing suicide prevention peer-led support programs, we also approached clinical and academic content experts and searched relevant internet sites including organisations known to be active in suicide prevention. See Additional file 1: Table S1 for a list of identified and screened websites and programs.

Search terms were developed relating to the three key concepts underpinning the literature review: suicide, peer support and lived experience. These were in alignment with our definition of lived experience peer-led support programs in suicide prevention. Alternative terms for peer support were developed and refined during iterative test searches. The final search strategy was developed using medical subject headings and free text words related to peer support and suicide prevention. Search terms were adjusted to fit the requirements of different databases. A complete list of search terms by database is available in Additional file 2: Table S2.

### Study selection

All records were imported into Endnote (version X8.2) and screened for inclusion in two stages. In the first stage, one researcher (AM) screened titles and abstracts for potential inclusion. In stage 2, the full texts of retrieved articles were screened independently by two researchers (AM and MS). Discrepancies were resolved in a meeting between the two researchers. Google Scholar records were screened first by title and abstract and then by full text by MS. WHO Clinical Trials Registry entries were screened by LR. Grey literature was retrieved by IO via internet search and consultations with experts in the area and then screened for inclusion in a team meeting by IO, LR, MS and AM.

Records were eligible if they included a peer-led support program with a focus on suicide prevention for people who experience suicidality. Peer supporters had to have lived experience of suicide; they could be community volunteers, people with similar experiences, or health professionals/health care staff if they had a lived experience of suicide and this informed their support role as peer supporter. Our review was not restricted to programs with matched lived experiences. There were no restrictions on the delivery mode of the programs; for example, programs could be delivered one-on-one, in group settings, as telephone support, online, at home, or in respite care.

We excluded records if programs were not delivered by people with a lived experience of suicide or where this could not be determined from the program description; were suicide bereavement programs; were capacity building or workforce training programs such as gatekeeper training, suicide awareness raising or suicide literacy programs; were focussed on improving mental health more broadly; or were a component of a multi-component intervention and not described separately within the larger program.

**Fig. 1:**
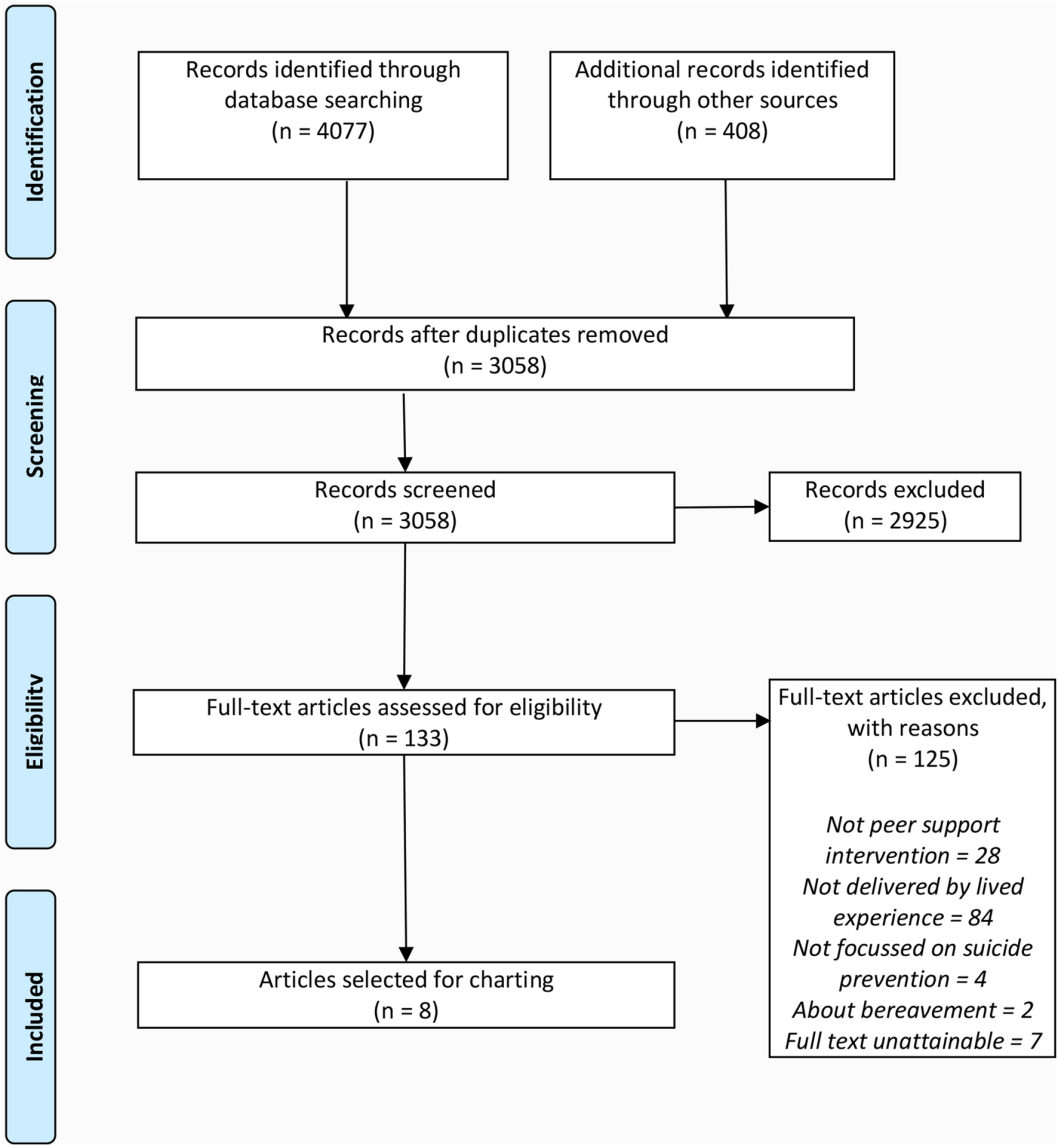
Flow diagram illustrating the literature search process

Articles in academic databases and Google Scholar had to be published between January 2000 and August 2019 and trials had to be registered on the WHO Clinical Trials Registry between 2013 and 2018. Academic databases were first searched on 14 June 2018. A second search of the academic databases was run on 29 August 2019 after the authors became aware of new evidence published since the original search. Google Scholar was searched on 21 June 2018 and the Trial Registry was searched on 7 September 2018. There was no time restriction on programs and records identified through expert consultation and web searches. We included any evaluation reports of eligible programs, irrespective of study design, setting, participant age, and publication language, so long as they could be translated into English. The Flow Diagram in Fig. 1 includes the number of records at each screening stage for all data searches and data sources combined.

Following these criteria, we identified 4077 records through electronic searches and 408 through searching other sources including grey literature, websites and expert consultations. Following removal of duplicates, 3058 records were screened by title and abstract information. This led to the exclusion of 2925 records. The remaining 133 records were read in full text by AM and MS who reached consensus on the final inclusion of 8 records according to the selection criteria. Of these 8 records, two referred to the same program, and were therefore summarised as one program in the results section.

### Charting the data

Records identified for final inclusion were extracted into a charting table which documented the following information: Reference, title and country of the program; program description including setting modality and lived experience mode; study aim and methodology; sample characteristics and key findings (if the program had been evaluated). Table 1 lists details on all identified programs.

**Table 1:** Description of included studies

Reference; program title; Country	Program description (setting, modality, lived experience etc.)	Study aim and methodology	Sample characteristics (where relevant)	Key findings (where relevant)
References: [29, 30] Title: Selbstmordforum.de (Translation: suicideforum.de) Country: Germany	*Setting:* Online messaging boards (forums); *Modality*: Group support; *Target group:* Users of online suicide forum *Lived experience:* 88% of study participants reported suicide ideation; 54% of participants had made at least one suicide attempt in their life. 28% had suicidal thoughts for the first time 1–3 years ago; 34% had suicidal thoughts more than 5 years ago for the first time; 12% never had suicidal thoughts.	*Aim:* Investigate whether the use of online suicide message boards is harmful or beneficial to people with suicidality. *Methodology:* Quantitative cross-sectional study; Convenience sample; online questionnaire of people using the online suicide message board “selbstmordforum.de”; Analysis of users’ socio-demographic characteristics, their motivations for participation, what content they shared, and what effect participation in online suicide messaging boards had on participants	164 participants; 50% male, 50% female; sample biased to young adults with 59% under 21 years and 88% under 30 years; 80% single; 67% students or in higher education settings	Online suicide forums do show signs of support and constructive help through conversations with other people with lived experience. Decrease in intensity of suicidal thoughts during the respondents’ participation in the suicide forum; 31% of participants say the decrease is due to their participation. Participation does not seem to increase help-seeking. Only 22% of respondents said they were more motivated to seek help. Motivation to use the forum was feeling understood and receiving comforting reactions.
Reference: [31] Title: Suicide messaging boards Country: Germany/Austria	*Setting:* Online messaging boards (forums); *Modality:* Group support; *Target group:* Users of online suicide messaging boards *Lived experience:* Suicidality of the online user was coded according to the content in the comments on the messaging board.	*Aim:* Identifying communication patterns that can be used to improve suicidality of participants on online suicide message boards. *Methodology:* Qualitative study; Secondary data analysis of a random selection of threads from 7 pre-identified suicide message boards (online forum); Thematic analysis of threads	401 threads from anti-suicide boards, 382 threads from neutral boards; 399 threads from pro-suicide boards	Constructive advice, active listening, collaborative problem solving, expression of sympathy, debunking the suicide myths, and provision of alternatives to suicide and positive stories of lived experience help to improve a participant’s suicidality.
Reference: [35] Program: Montgomery County Emergency Service—Peer specialist support Country: USA	*Setting:* Hospital, patient care; *Modality:* Mixed mode, including one-on-one peer support; two part-time peer specialists provide services to in-patients, families and community members including support groups, one-on-one counseling, training and capacity building in the community. *Target group:* Patients of a psychiatric hospital and people in crisis support *Lived experience:* Peer specialists have past experience of suicide and received peer support and recovery training.	*Aim:* Description of suicide prevention service within a hospital—in-patient care	N/A.	N/A.
Reference: [34] Program: Health Intervention Training—Mutual Aid Network (HIT-MAN) Country: China	*Setting:* Schools; *Modality:* Peer group support based on a social network approach; Objectives are to encourage students to support and, in some cases, refer their friends who have been thinking about suicide or showing very poor self-esteem and signs of depression to a trusted adult (who will also be part of the HIT-MAN training network). *Target group:* School students (adolescents), teachers and school communities *Lived experience:* Students in peer groups have lived experience of mental health problems which are seen as early warning signs of the trajectory to suicide.	*Aim:* Description of a suicide prevention program that helps students to identify problems of extreme stress, anxiety and suicidal feelings in their peers, including training and peer support groups.	N/A.	N/A.
Reference: [32] Program: Peers for Valued Living (PREVAIL) Country: USA	*Setting:* Inpatient psychiatric unit and post discharge care *Modality:* One-on-one peer support; Peer specialists first meet patients in inpatient unit and then provide support for 12 weeks after discharge (max 16 meetings); flexible meeting arrangements (frequency and duration); *Target group:* Patients admitted to an inpatient psychiatry due to suicidality *Lived experience:* Peer specialists had lived experience of serious suicidal thoughts or behaviour.	*Aim:* Development and pilot testing of a peer specialist intervention to reduce suicide risk, *Methodology:* Pilot randomised controlled trial; randomisation of participants to normal care and PREVAIL peer support intervention; Semi-structured interviews to capture participant experiences and feedback	70 adult patients (age 18 or older) from two inpatient psychiatric units; patients had to have history of suicidal ideation or attempt; patients were excluded from participation if they showed unstable psychosis, cognitive impairment, severe personality disorder, invasive therapy. 34 were allocated to the peer support arm and 36 received usual care.	Feasibility and acceptability for the program was assessed by collecting quantitative and qualitative data on peer specialist performance and training. No data was provided on the efficacy of the program to reduce suicidality due to lack of power in the trial.
Reference: [28] Program: Alternatives to Suicide through the Western Mass Recovery Learning Community (RLC) Country: USA	*Setting:* Community led intervention; *Modality:* Peer support group for people who experience suicidality; underpinned by the key principles of Validation + Curiosity + Vulnerability + Community; It creates conditions that support recovery at both the individual and community level through trauma-sensitive peer supports and the development of a regional network. *Target group:* People who experience suicidality (not bereavement) *Lived experience:* Peer support groups are open to anyone with lived experience of suicidal thoughts or attempts; Leaders who struggled with thoughts of suicide or suicide attempts and who wanted to support others.	*Aim:* Description of the Alternative to Suicide (ATS) peer support program, an innovative peer-led suicide prevention program from the Western Massachusetts Recovery Learning Community (RLC). *Methodology:* Internal feedback survey with attendees of support groups.	not available	Early findings from an internal feedback survey with attendees of the support groups: attendees felt most strongly that attending the groups was helpful because they could talk freely; attending had improved at least one area in attendees’ lives; increased sense of community and increased understanding on why suicidal thoughts may come up were areas with greatest improvement.
References: [33, 36, 37] Program: The Way Back Support Service—Peer CARE Companion Program Country: Australia	*Setting:* Community-based intervention; *Modality:* One-on-one support; *Target group:* People with lived experience of suicide (suicidal crisis or after a suicide attempt) *Lived experience:* Peer support is customised depending on whether peers are to be supporting people at risk. Lived experience of the peer care companion will be matched with the person in need.	*Aim:* Description of the Peer CARE Companion program which provides peer support to people with lived experience of suicide or bereaved by suicide.	N/A.	N/A.

### Collating and summarising findings

The information in the charting table was then synthesised in accordance with the two research questions of this paper. Due to the variation in study design and the absence of evaluation data for many identified programs we focussed on a narrative summary of studies. First, we provide an overview of the types of peer support suicide prevention programs (research question 1) by briefly summarising programs by their setting, modality and the role of lived experience. Secondly, for those programs that had been evaluated we characterise the study aim and methodology and discuss key findings regarding their effectiveness for suicide prevention (research question 2) (Table 1).

## Results

### Types of suicide prevention peer support programs

Our search identified eight records that fulfilled the inclusion criteria. These described seven programs that were from four different countries: USA [3], Germany [2], China [1], and Australia [1], and which varied in setting and design (see Table 1). Of the seven programs, four included an evaluation component [28–32] and three provided descriptive accounts of a program [33–35]. Four peer support programs provided group support [28–31, 34], two were designed to deliver one-on-one support [32, 33] and one program included mixed modes of support [35]. Regarding the program settings, two programs were delivered in clinical settings [32, 35], two in the community [28, 33], two online [29–31] and one in schools [34].

Salvatore [35] presented a mixed mode peer support program within a psychiatric hospital. It was offered to patients of the hospital and their families. The hospital employed two peer support staff to deliver one-on-one peer support as well as group support. While this program has been implemented as part of the Montgomery County Emergency Service, evaluation data on its effectiveness has yet to be published. The second program designed for patients in clinical settings was the Peers for Valued Life (PREVAIL) program [32]. This program is a one-on-one support service for people who had attempted suicide and were patients of a psychiatric ward. Patients were teamed up with a peer specialist with lived experience of suicide and weekly meetings were held for up to 12 weeks after discharge from the ward. Peer specialists received training on risk assessment, using suicide prevention tools, communication and relationship building and motivational interviewing. In situations of acute risk clinicians were contacted. The aim of this program was to reduce suicide risk post-discharge from a psychiatric ward.

The two community-based suicide prevention programs were designed to provide support to those in crisis or experiencing suicidal ideation. Firstly, the Alternatives to Suicide program (USA) runs peer support groups for people experiencing suicidality [28]. Open group discussions are facilitated by trained peer supporters to enable conversations around the reasons and factors that may have contributed to someone wanting to die. Reflective of the key principles of Validation, Curiosity, Vulnerability and Community the conversations are non-judgemental and free of boundaries. Groups provide a safe and comfortable space to talk and focus on offering a non-clinical environment to build trust. Secondly, the Peer CARE Companion Program (Australia) offered through the Way Back Support Service is a new program which is currently being trialled [33, 36, 37]. It is directed at supporting people with a lived experience of suicide (experiencing a suicidal crisis or after a suicide attempt) in one-on-one peer support settings. The program was developed through a collaboration between three mental health organisations in Australia (Beyond Blue, New Horizons and Roses in the Ocean). Two trials and a consultation process including people with lived experience in 2017 and 2018 led to a revision of the program and the results of a second trial are yet to be released.

Two programs were set online using data from online messaging boards [29–31]. Both aimed at better understanding the benefits and risks of participation to people experiencing suicidality by looking at the effect that using online messaging boards has on participants. These online messaging boards are best described as informal group support interventions. While meaning to support people who experience suicidality, due to their open entry format they allow both people with lived experience and non-suicidal people to participate. The content is participant/online user driven with limited control for quality and safety for people at risk.

Finally, a school-based program aimed at early detection of at-risk youth in Chinese schools, offered peer group support sessions led by students and supported by teachers and the school community [34]. Students at risk were identified by peers or teachers and invited to participate in student-led support groups that met regularly. Teachers visited the groups monthly to help address any issues if needed. Group membership was voluntary and group leadership rotated. Group members were taught how to recognise unhappy and depressive behaviour in peers. Group leaders reported to the teacher and were able to refer students further if concerned.

With the exception of the school-based peer support program, which broadened its scope to include mood, depression and self-esteem as early warning indicators of suicidality, all programs were specifically aimed at supporting people with a lived experience of suicide. It was however decided to include the school based intervention as its overall goal was described as reducing suicide risk in youth.

### Effectiveness of suicide prevention peer support programs

Three of the seven identified programs contained evaluation data; two were quantitative studies [28–30], one was qualitative [31] (see Table 1).

The Alternatives to Suicide program reported early findings from internal feedback surveys with attendees of the support groups [28]. Findings indicate that attending the groups was perceived as helpful as participants felt that they could talk freely about their experiences. Attending the groups had improved at least one area in attendees’ lives. Areas of greatest improvement were increased sense of community and a better understanding of why suicidal thoughts may come up.

Kral and Eichenberg found that participation in online peer support forums decreased the intensity of suicidal thoughts [29, 30]. The authors collected data from participants of the online forums via an online survey. Thirty-one percent of participants self-reported a decrease in intensity of suicidal thoughts as a result of their participation in the messaging boards. While 22% of respondents said they were more motivated to seek professional help, using suicide messaging boards did not increase help-seeking outside the forums. The main motivation for using online forums was for emotional support, to feel understood and receive comforting reactions.

In a qualitative analysis of threads from a suicide online forum Niederkrotenthaler and colleagues [31] found that participation in this forum can help to improve a person’s suicidality. The authors downloaded threads from seven pre-identified suicide message boards and thematically analysed a random selection of these threads. Several communicative strategies were associated with psychological improvements in online forum participants; these were receiving constructive advice, being actively listened to, receiving sympathy for one’s struggle, and provision of alternatives to suicide by other members of the forum.

While the Pilot Randomised Controlled Trial of the PREVAIL program did assess the programs feasibility and acceptability by collecting quantitative and qualitative data on peer specialist performance, this trial was unable to assess the programs efficacy in reducing suicidality in participants due lack of power [32].

## Discussion

Peer-led support programs are increasing in mental health and suicide prevention and are seen as worthy additions to conventional clinical care and alternative support options for community care [38]. While there is evidence for the effectiveness of peer support programs for people with severe mental illness and also for people bereaved by suicide [14, 18], only little and mostly anecdotal data has been published on peer-led support programs with a focus on reducing suicidality. While Government organisations and peak agencies are calling for an inclusion of peer-led support into standard mental health care models, the evidence on what these support services look like, how they can be integrated into conventional care and how effective they are as a stand-alone service is lacking. This led to believe that research in this space is still in its infancy and therefore warrants further attention.

This study is the first scoping review of published literature on peer support programs for people experiencing suicidality. We systematically searched academic databases, grey literature databases, searched the web and consulted experts in the field, strictly focussing on peer-led support programs that set out to reduce suicidality in participants. We strictly excluded programs with a broader focus on mental health and also excluded bereavement programs. Our search identified 8 records accounting for 7 programs that focussed on peer-led support programs in suicide prevention. The 7 eligible programs employed a range of different designs and included a variety of settings (schools, communities, rural and online). Only three programs provided evaluation data, and this data was descriptive on all accounts. This small number of eligible programs highlights a general scarcity of publications on peer-led suicide prevention programs and their evaluation. While the little data available indicates some positive and promising results for peer-led support in suicide prevention, it remains anecdotal at this stage. Despite the increased recognition of peer-led support programs this review highlights that the evidence gap on effective designs and efficacy of programs persists.

Our findings hint at the potential for online forums as a support hub for people with lived experience of suicide and the potential for increased research for peer-led support in this setting, keeping in mind the risk that these unmoderated environments can carry. During the screening of records we identified a large number of community driven initiatives, yet none of them had been evaluated and many focussed on awareness raising and training of support workers and were therefore not included here. In essence there is an evidence gap for peer-led community-based suicide prevention programs regarding their effectiveness to reduce suicidality in the community.

On an exciting note, our consultations with experts suggests there are signs for new peer-led community-based peer support programs to be developed and future evaluations of some of these programs are planned. The pilot trial of the PREVAIL peer support program shows that it is possible and feasible to integrate peer support into the care program for people who experience suicidality, yet formal evaluation of these kinds of programs is needed to determine their effectiveness to reduce suicidality [32]. Since the potential for peer-led support in suicide prevention has increasingly been acknowledged by policy makers [39] we anticipate that this in turn will positively influence program development and evaluation in the future and that we will continue to expand our knowledge and understanding of peer-led suicide prevention.

### Challenges and limitations

In screening the literature, we identified that 84 records had to be excluded in the full-text review stage due to either not providing enough information on the nature of the lived experience of peer supporter workers or because lived experience was defined more broadly and not specific to suicidality. For example, it was frequently unclear if a peer support program was in fact peer-led or led by a clinician, and when the program was peer-led it did not specify if the peer supporter had lived experience of suicide or whether this was defined more broadly in the context of mental health. It is possible that the lack of clarity in the definition of peer support may have led to the exclusion of otherwise eligible programs during the screening process of this review.

Further, we found that in some programs the definition of peers was not aligned with our selection criteria. Some programs described their intervention as peer group support, yet the group was led by a health professional [40]. Others had a peer supporter as co-facilitator alongside a leading health professional, therefore not qualifying as a peer-led program [41]. In particular, school-based programs working with students tended to select peers on the basis of age or belonging to the same social group but did not make suicidal experiences part of the condition for becoming a peer supporter [42, 43].

Despite the effort that has gone into defining peer support and lived experience in the context of suicide prevention in recent years [2, 10], this seemingly has not yet translated into research designs and publications. The findings from this review highlight that authors follow varying definitions for peer-led support and often fail to provide adequate detail in the description of their program about what constitutes peer-led support in their respective program. This limits our understanding of the nature of peer support within existing programs and ultimately affects what implications we can draw from existing literature on the effectiveness of peer-led support suicide prevention programs.

### Implications for future research, policy and practice

To advance knowledge on peer-led support programs in suicide prevention we suggest a few areas for future investment. Firstly, the development of a framework for standard reporting on peer support initiatives would greatly improve our understanding of the breadth and depth of current peer support programs [44]. In addition, improved quality of reporting on peer support roles in suicide prevention programs would help to clarify the underlying definition of peer support. Secondly, we need high-quality evaluations of peer support suicide prevention programs and of peer-led components within larger programs to better understand their effectiveness on participant health across different settings and delivery modes and to allow for comprehensive systematic reviews and meta-analysis in the future. This evidence can help enhance our efforts to better integrate peer-led support with conventional crisis support and find mutual benefit in both. Thirdly, while peer support is generally accepted as a positive addition to care by legislative bodies, we currently lack models for the efficient and effective integration of these programs alongside conventional care [38, 45]. Addressing this issue would facilitate peer support to become a care component in its own merit.

It should also be noted what is already known about the positive effects of peer-led support in other related areas. While the knowledge is scarce on peer-led suicide prevention programs, it could be beneficial to revisit evidence from mental health peer support and investigate whether similar approaches could be adapted to suicide prevention. This practical approach could then be subject to further testing and refining to cater to specific needs in suicide prevention.

## Conclusion

While peer support programs are seeing greater support in the community, in health care and by policy makers, very little is known about their effectiveness in the context of suicide prevention. This scoping review set out to review the evidence available to date. Yet, we identified very few peer-led support suicide prevention programs and even fewer evaluations. To improve our understanding in this field we encourage greater clarity in the reporting of key program characteristics and components and highlight the need for formal program evaluation. This will greatly assist in creating a vital evidence base to inform future program development and implementation which is much needed in this space.

## Supplementary information

**Additional file 1: Table S1.** Search strategies by data bases.

**Additional file 2: Table S2:** Programs identified through web search and expert consultations and screened for inclusion.

## Data Availability

All data generated or analysed during this study are included in this published article and its supplementary information files. The literature compiled through this search is available through publications and the internet.
