# Supplementary material for: Lived experience peer support programs for suicide prevention: a systematic scoping review
Source: Int J Ment Health Syst. 2020 Aug 12;14:65. doi: 10.1186/s13033-020-00396-1 (PMC7425132; doi:10.1186/s13033-020-00396-1)
Supplement: Supplementary file 1 — Additional file 1: Table S1. Search strategies by data bases. [file 13033_2020_396_MOESM1_ESM.docx]

**Additional file 1**

**Table S1: Search strategies by data bases**

| **Academic databases - 14 June 2018 and 29 August 2019** | |
| --- | --- |
| **Medline (PubMed)** | (self-help groups[mesh] OR volunteers[mesh] OR mentors[mesh] OR peer[TIAB] OR consumer[TIAB] OR volunteer[TIAB] OR service-user[TIAB] OR "lived experience"[TIAB] OR support worker[TIAB] OR respite[TIAB]) AND (Suicide[Mesh:NoExp] OR "Suicide, Attempted"[Mesh] OR "Suicidal Ideation"[Mesh] OR suicid*[TIAB]) |
| **PsycINFO** | (peer counseling/ OR peers/ OR support groups/ OR outreach programs/ OR self-help techniques/ OR volunteers/ OR mentor/ OR (peer OR consumer OR volunteer OR service-user OR "lived experience" OR support worker OR respite).tw) AND (suicide/ OR suicidal ideation/ OR attempted suicide/ OR suicidology/ OR suicide prevention/ OR suicide prevention centers/ OR suicid*.tw) |
| **Embase** | (peer counseling/ OR volunteer/ OR self help/ OR support group/ OR mentor/ OR (peer OR consumer OR volunteer OR service-user OR "lived experience" OR support worker OR respite.tw) AND (suicide/ OR suicidal ideation/ OR suicide attempt/ OR suicid*.tw) |
| **Other sources** | |
| **Google Scholar - 21 June 2018** | allintitle: suicide OR suicidal OR suicidology "peer counseling" OR “peer counselling” OR volunteer OR "self help" OR "support group" OR mentor OR peer OR consumer OR "service-user" OR "lived experience" OR "support worker" OR respite |
| **WHO Trial Registry – 7 Sept. 2018** | suicid* AND (peer OR volunteer OR "support group" OR mentor OR consumer OR "lived experience" OR respite) |
