# Supplementary material for: Lived experience peer support programs for suicide prevention: a systematic scoping review
Source: Int J Ment Health Syst. 2020 Aug 12;14:65. doi: 10.1186/s13033-020-00396-1 (PMC7425132; doi:10.1186/s13033-020-00396-1)
Supplement: Supplementary file 2 — Additional file 2: Table S2: Programs identified through web search and expert consultations and screened for inclusion. [file 13033_2020_396_MOESM2_ESM.docx]

**Additional file 2**

**Table S2: Programs identified through web search and expert consultations and screened for inclusion**

| **Program Name** | **References/websites** |
| --- | --- |
| Conversations for Life | www.connetica.com.au |
| Yarns for Life | www.connetica.com.au |
| Strengthen for Life | www.connetica.com.au |
| Alternatives to Suicide | http://www.westernmassrlc.org/alternatives-to-suicide |
| Dealing with Crisis | www.peerzone.info https://www.peerzone.info/workshops/9 |
| Livingworks /ASIST/Safe TALK | www.livingworks.com.au |
| "Hope for Life" & Bereavement program" | www.salvos.org.au |
| Wesley LifeForce program | www.wesleymission.org.au |
| SALT: See, Ask, Listen, Tell | <https://www.wesleymission.org.au/> wesleylifeforceservicefinder/salt-strategy/ |
| The Safe Haven Café drop in Centre at St. Vincent's Hosp, Melbourne | www.stvincentsmelbourne.blog |
| Everymind | www.everymind.org.au |
| The Way Back Support Service NSW | https://everymind.org.au/programs/twb-support-service-nsw |
| Wellways (formerly Mental Illness Fellowship of Aust (Vic) | https://www.wellways.org/ |
| QPR Institute Australia | www.qprinstituteaustralia.com.au |
| Neami National - Assess & Respond to Suicide | https://www.neaminational.org.au/ |
| Place of Calm | www.acalmplace-reiki.melbourne |
| Light House Foundation | www.lighthousefoundation.org.au |
| Mates 4 Mates | www.mates4mates.org |
| Flourish - formerly Richmond PRA | www.flourishaustralia.org.au |
| CALM - The Voice of Education in Suicide | via www.lifeinmindaustralia.com.au |
| Community action for the Prevention of Suicide (CAPS)Inc | www.caps.org.au |
| Mates in Construction | www.mic.org.au |
| Mates in Mining | www.mic.org.au |
| Men's Sheds | www.mensshed.org |
| MindWorx Mood Support group - Geelong | www.mindworx.org.au |
| Male Suicide Prevention Australia | www.malesuicidepreventionaustralia.com.au |
| NewAccess | www.beyondblue.org.au |
| Young Men's project | www.youngmensproject.com.au |
| Inco-link (Victoria) | www.incolink.org.au |
| Lifeline | www.lifeline.org.au |
| ECLIPSE Support group | www.lifelinemacarthurgroup |
| Compeer | www.vinnies.org.au |
| The Streat | www.streat.com.au |
| BrookRed Peer Support | www.brookred.org.au/peer-support |
| BrookRed Red House | www.brookred.org.au/red-house |
| MindfullAus | www.mindfullaus.org.au |
| Mountains of Hope Network |  |
| Roses in The Ocean | www.rosesintheocean.org.au |
| WRAP® (Wellness Recovery Action Plan) | www.copelandcenter.com |
| Mental Health Association of Oregon (MHAO) | www.mhaoforegon.org |
| Kevin Briggs | www.pivotal-points.com |
| Kevin Hines | www.kevinhinesstory.com |
| Sally Spencer | www.sallyspencerthomas.com |
| Carson J Spencer Foundation | www.constructionworkingminds.org |
| United Suicide Survivors International | www.unitesurvivors.org |
| American Association of Suicidology | www.suicidepreventionlifeline.org |
| The National Suicide Prevention Lifeline – l Live through this | www.livethroughthis.org |
| Substance abuse & MH Services Administration - SAMHSA | www.sprc.org |
| Buddy-to-buddy: Volunteer Veteran peer support program | https://m-span.org/buddy/ |
| Breaking the Silence (USA) | www.breakingthesilence.org.il |
| Counseling on Access to Lethal Means (CALM) (USA) | www.sprc.org |
| Caring CONTACTS | www.caringcontact.org |
| Canadian Mental Health Association | https://cmha.ca/ |
| The Mental Health Commission of Canada | https://www.mentalhealthcommission.ca/English |
| Psychiatric Survivor of Ottawa | https://www.pso-ottawa.ca/ |
| Dude’s Club (Canada) | https://www.dudesclub.ca/ |
| Simpson, A, Quigley, J, Henry, S.J., & Hall, C (2014) | https://www.mindout.org.uk/get-support/suicide-prevention/ |
| Campaign Against Living Miserably (CALM) (UK) | https://www.prevent-suicide.org.uk/index.html |
| Men’s SHARE (Suicide, Harm, Awareness, Recovery and Empathy) (Scotland) | www.health-in-mind.org.uk |
| Scottish recovery network | www.scottishrecovery.net |
